# The formation of patient trust and its transference to online health services: the case of a Dutch online patient portal for rehabilitation care

**DOI:** 10.1186/s12911-021-01552-4

**Published:** 2021-06-12

**Authors:** Lex van Velsen, Ina Flierman, Monique Tabak

**Affiliations:** 1 eHealth Group, Roessingh Research and Development, P.O. Box 310, 7500 AH Enschede, the Netherlands; 2 Biomedical Signals and Systems Group, University of Twente, Enschede, the Netherlands; 3 Roessingh Center for Rehabilitation, Enschede, the Netherlands

**Keywords:** eHealth, Trust, Trust transference, Rehabilitation, Medical services, Online patient portal

## Abstract

**Background:**

Trust is widely recognized as a crucial factor in successful physician–patient communication and patient engagement in treatment. However, with the rise of eHealth technologies, such as online patient portals, the role of trust and the factors that influence it need to be reconsidered. In this study, we aim to identify the factors that contribute to trust in an eHealth service and we aim to identify the consequences of trust in an eHealth service in terms of use.

**Methods:**

The Patient Trust Assessment Tool was provided to new outpatients of a rehabilitation center in the Netherlands, that were expected to use the center’s online patient portal. Via this tool, we assessed five trust-related factors. This data was supplemented by questions about demographics (age, gender, rehabilitation treatment) and data about use (number of sessions, total time spent in sessions), derived from data logs. Data was analyzed via Partial Least Squares Structural Equation Modelling.

**Results:**

In total, 93 patients participated in the study. Out of these participants, 61 used the portal at least once. The measurement model was considered good. Trust in the organization was found to affect trust in the care team (β = .63), trust in the care team affected trust in the treatment (β = .60). Both, trust in the care team and trust in the treatment influenced trust in the technology (β = .42 and .30, respectively). Trust in the technology affected the holistic concept trust in the service (β = .78). This holistic trust in the service finally, did not affect use.

**Conclusions:**

This study shows that the formation of this trust is not unidimensional, but consists of different, separate factors (trust in the care organization, trust in the care team and trust in the treatment). Trust transfer does take place from offline to online health services. However, trust in the service does not directly affect the use of the eHealth technology.

**Supplementary Information:**

The online version contains supplementary material available at 10.1186/s12911-021-01552-4.

## Background

Trust is widely recognized as a crucial factor in successful physician–patient communication and patient engagement in treatment [1, 2]. However, with the rise of eHealth technologies, such as online patient portals, the role of trust and the factors that influence it need to be reconsidered and different questions arise. Which factors affect trust in eHealth? And how does trust in an eHealth service affect use? While quite some research has focussed on patient’s trust in online health information, only little research has delved into these questions in the context of eHealth services. For this study, trust can be defined as “an individual’s belief in the competence, dependability, and security of the [online health service] under conditions of risk” [3 page 60]. In a series of focus groups with patients and healthcare professionals, Van Velsen et al. [4] uncovered that patient trust in an online patient portal is made up by several factors: Trust in the organization providing the online patient portal, trust in the patient’s care team, patient trust in his/her treatment, and trust in the technology itself. Lyles and colleagues identified that trust in online patient portals for patients with Diabetes is related to the end-user’s race and age [5]. It is still unclear, however, how the factors that make up trust relate to each other and how they influence the use of eHealth services.

A large body of literature on the consequences of trusting a digital service can be found for commercial services. Here, trust in the online service has been found to increase perceived usefulness, ease of use, customer satisfaction, the intention to make transactions, and to negate the perceived risks involved (e.g., [6–8]). A starting point for increasing our understanding of trust in online health services may be the concept of trust transference. Trust transference presumes that when there are two similar entities (e.g., a hospital and the hospital’s online patient portal), the trust that a person has in the entity that is known (here: the hospital) is transferred to a new, related entity (the online patient portal that the patient uses for the first time) [9]. This theory has been proven in different (mostly commercial) settings, such as from physical stores to their online counterpart [10] and from physical to online banking [11]. Within traditional, offline healthcare services, trust transfer has been observed, where trust in one hospital has been found to be transferrable to an allied hospital [12]. In a recent study among older adults, trust in offline health services (the trust in face-to-face consults in a hospital) was found to positively affect trust in mobile health services, which affected, on its turn, the intention to use these services [13]. In addition, trust in the care professional has been found to affect perceptions of an online patient portal [14].

The benefits of trust in offline health services have been widely documented. For example, trust in a primary care physician was found to increase patient satisfaction and loyalty to this healthcare provider [1]. Trust in a medical specialist turned out to lead to better communication and medical decision making, while it decreases patient fear and improves patient adherence to treatment [15]. And finally, trust in nurses can ensure a patient in times of uncertainty [16]. Studies that investigate the benefits of trust in eHealth services, however, are scarce. Beldad and Hegner [17] found that trust increases the perceived usefulness of a mobile fitness application, which in turn increases the intention to use. Klein [18] studied the formation of trust and its consequences for an online patient-physician communication service and found that trust in the healthcare provider and trust in the vendor of the technology positively affect the intention to use the service.

In this study, we aim to identify the factors that make up trust in an eHealth service (in this case an online patient portal for rehabilitation care), using the concept of trust transference. Next, we aim to identify the consequences of trust in an eHealth service in terms of use. While many (popular) articles on eHealth (e.g., [19–21]) claim that trust in an eHealth service is paramount for its success, the way in which this trust comes about, and the manner in which it should be dealt with in implementation plans is largely unknown. The results from this study will allow eHealth developers, implementation managers and policy makers to make better informed decisions about the way in which they should implement eHealth in an organization. This article is organized as follows. In the Method section, we present our research model and data collection and data analyses approach. Next, we present the Results, followed by a Discussion that includes an overview of the limitations of this study. In our Conclusions section, finally, we discuss our findings and explore avenues for future research.

## Method

### Research model

**Fig. 1:**
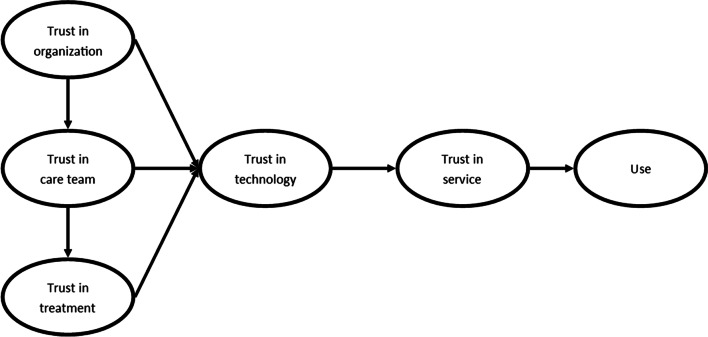
Research model

In order to determine which factors make up trust in an eHealth service and its effect on the use of an eHealth service, we posit the research model that is displayed in Fig. 1.

Trust in a health care setting (e.g., a hospital or primary care practice) has been found to affect patient trust in the physician that operates within this organization [22, 23]. Trust in a care professional, on its turn, has been positively associated with trust in the treatment, prescribed by this care professional [24]. Translated to the context of rehabilitation care, where treatment is offered by a multidisciplinary team of care professionals, this suggests that trust in the care team positively affects trust in the treatment. Following the concept of trust transference (whereby trust in a known entity is transferred to a related but unknown entity), we then hypothesize that all three factors that determine trust in offline care (trust in the organization, care team, and treatment), positively influence trust in the technology that is introduced as part of the total care package. Then, in line with a result from a focus group study that explored the coming about of trust in an online patient portal for rehabilitation care [4], we presume that trust in the technology (which focuses on trust related to direct interaction with the technology) will affect an overall sense of trust: Trust in the service. This is a holistic concept that encompasses the trust in an eHealth service on an abstract level, in contrast to the previous trust factors, which focus on a specific aspect of trust. Following [25], finally, in which a literature review indicated that trust in an eHealth service may affect use, the overall sense of trust is hypothesized to positively influence use.

### Data collection

Data on trust-related factors was collected by administering the PAtient Trust Assessment Tool (PATAT) [26]. The PATAT is a trust benchmarking survey and, currently, the only instrument that provides a comprehensive measurement of trust in an eHealth service and the underlying trust concepts. The instrument is based on a focus group study [4] in which relevant trust factors were elicited. The survey has been validated in the context of anticoagulation management and was found to have good psychometric qualities and to explain a high degree of variance (R^2^ = 0.68) [26]. It includes 5 factors: Trust in the care organization providing the eHealth service, trust in the treatment, trust in the care professional, trust in the technology, and finally a holistic factor focused on trust in the eHealth service. Each factor is assessed via 5 statements and five-point Likert scales (except for Trust in the care professional, which is assessed via 4 statements). These Likert scales range from totally disagree (1) to totally agree (5). Use was operationalized by assessing the number of sessions each patient started on the portal and the time they spent on the portal (derived from data logs).

New outpatients of a rehabilitation center in the Netherlands were invited to take part in the study. The center is one of the largest centers for rehabilitation in the Netherlands and is specialized in all aspects of specialist rehabilitation care. The center employs about 600 people and treated 584 inpatients and 2.717 patients via the policlinic in 2019 [27]. The participating outpatients took part in a rehabilitation program for lung conditions, oncology, or chronic pain. During such a program, the patients receive treatment from different professionals, such as physical therapists, occupational therapists, and psychologists. When first visiting the center for their treatment, these patients received information about their treatment and instructions for using the center’s online patient portal: Telerevalidatie. This online patient portal offers the following main functionalities:

- (health) information and education,
- a personalized exercise program (supported by video instructions),
- surveys for monitoring health and preparing appointments,
- and a module that allows patients to communicate with their care team via an internal e-mail system.

In between data collection and the publication of this article, Telerevalidatie has redesigned its interface, expanded its exercise catalogue and was made interoperable with the leading electronic patient record in Dutch rehabilitation care. At the moment, Telerevalidatie is the most-widely used online patient portal for rehabilitation care in the Netherlands; the online patient portal is being used by 12 Dutch centers for rehabilitation [28]. At the time of the study, the portal provided the key features of a modern eHealth service and was an integral part of the rehabilitation treatment in the rehabilitation center. Nonetheless, use of the portal was elective. Therefore, it was a very appropriate case to study in relation to trust and use; patients had every reason to use it, but were in no way forced to. More information about Telerevalidatie can be found at https://www.telerevalidatie.nl.

After their first visit, all new patients received an invitation via email to complete the PATAT in an online survey tool. Since the patients were invited by different physical therapists, we were unable to monitor the number of invitations that were sent out, and thus, to calculate a response rate. The PATAT items were adapted so that they would align with the context. This means that for the items questioning trust in the care organization, the name of the center for rehabilitation was inserted; for the items questioning trust in the care professional the term ‘doctor’ was replaced by ‘care team’ (e.g., I always follow my care team’s advice); and the term ‘technology’ was replaced by ‘the portal’ (e.g., When I use the portal, I am in control). All items can be found in Additional file 1: Measurement items. The items were supplemented by questions about gender, age, and to which rehabilitation group the patient belonged. Additionally, we asked the participants for their internal patient rehabilitation ID. Using this ID, we linked the data logs that were collected for this patient for the time span of a year, starting at the date of the instruction session. These data logs contained a time stamp for the beginning and end of each session on the portal. Data collection started in October 2015 and ended in December 2017. While some time has passed between data collection and publication of this work, the outcomes are still relevant as the online patient portal and its integration in rehabilitation care are still the state of the art.

Due to the nature of this research (portal use was part of the regular care process) and the voluntary nature of completing the survey, this study did not require formal ethical approval, according to Dutch law. All participants provided informed consent at the start of the online survey (for the survey data) or was assumed by use of the portal (for the log data).

### Data analyses

Demographic data was analyzed on a descriptive basis. The number of sessions and session length (in seconds) were the main outcome parameters for the data log analyses. Following [29], we used the number of sessions with the portal and the total time spent on the portal to form a general assessment of the factor Use. We considered a session to be a true session if it lasted at least 20 s. The portal provides functionality (like exercising, completing monitoring forms) that take some time to complete. Therefore, a cut-off of 20 s was assumed, to distinguish the sessions in which people only logged in and then stopped using the portal immediately, from the sessions in which functionalities for the benefit of treatment were really used. This period of 20 s was based upon the authors’ experience with the online patient portal. The research model was tested via Partial Least Squares Structural Equation Modelling (PLS-SEM) using Smart PLS 3.0 [30] and the guidelines by Hair Jr et al. [31]. At first, we assessed the measurement model, by determining outer loadings of the items, as well as cross-loadings. Then, we determined the reliability of the resulting measurement scales by assessing the composite reliability score, the Average Variance Extracted (AVE), and Cronbach’s alpha for each factor. After that we checked the measurement model for multicollinearity (via Variance Inflation Factor (VIF) scores) and determined whether the contribution of each item towards its construct is significantly greater than 0. Additionally, we calculated the correlations among the latent variables and compared them to the AVE root square values. The second step was to test the causal model and determine the effect sizes (*f*^2^). Given our research model, in which there is a maximum of three factors influencing another factor (trust in the organization, care team and treatment are hypothesized to affect trust in the technology), our desire to apply a significance level of 5% for testing the causal relations between factors (path coefficients) and 80% statistical power, a sample size of at least 59 participants would be necessary to detect an R^2^ of at least 0.25 for any dependent variable [31]. We have opted for 0.25 as this amount of explained variance means that the factor is relevant for the model.

## Results

### Demographics

In total, 93 patients from the outpatient rehabilitation programs participated in the study. Forty of them (43.0%) were male, while 53 participants (57.0%) were female. Their ages ranged from 28 to 76 years, with an average age of 58.07 years (SD = 9.47 years) and a mode of 64 years. Eighty-one persons indicated the rehabilitation program they participated in: 48 persons (51.6%) took part in the lung rehabilitation program, 28 persons (30.1%) took part in the oncological rehabilitation program, and 5 persons (5.4%) in the chronic pain rehabilitation program.

**Fig. 2:**
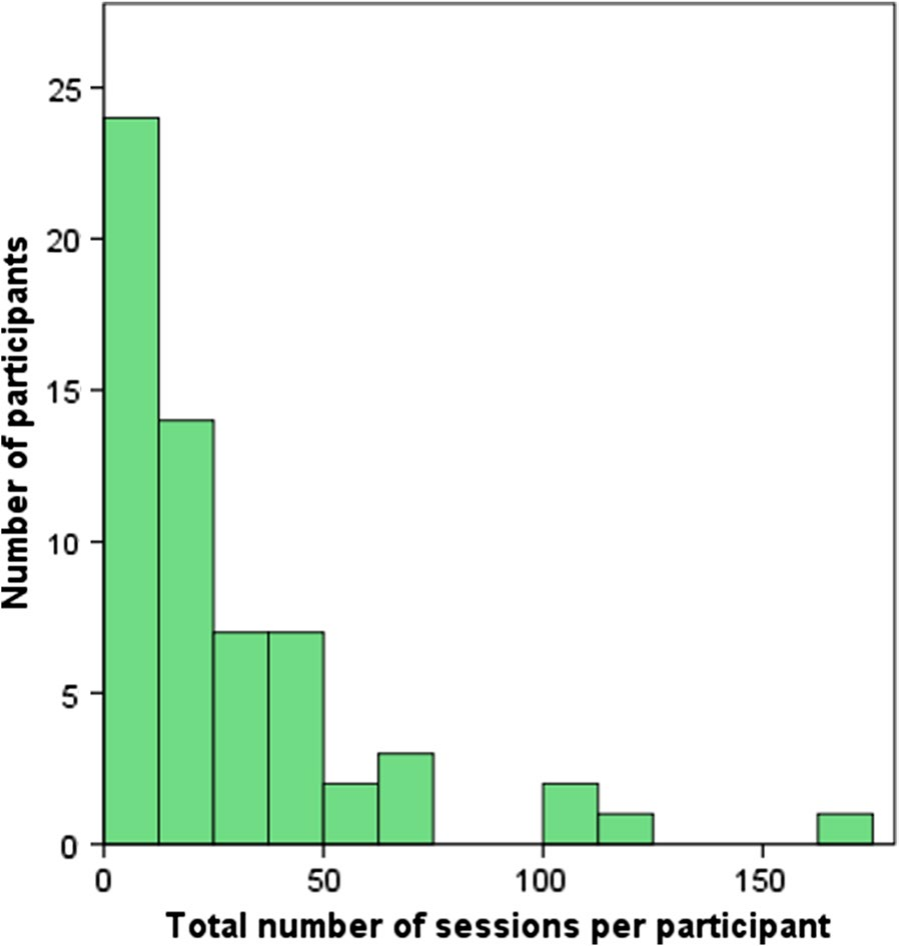
Total number of sessions per participant (n = 61)

Out of the 93 participants, 61 used the portal at least once, with a total of 1750 sessions within an average period of 74 days. Figure 2 shows the total number of sessions per participant and how these totals are distributed over the participant sample. The average number of sessions was 28.69. Of those sessions, 61.26% (n = 1072) lasted longer than 20 s, and can be considered sessions in which the participants used portal functionalities.

### Measurement model

As a first step, we determined the quality of our measurement model, and the items that are supposed to measure them. The PATAT measurement tool was previously validated, albeit for a different setting [26]. This validation can therefore be seen as a replication. Since Use was operationalized by means of two manifest variables (number of sessions and total time spent using the technology), this factor was not included in these analyses. The first step we took was to assess the convergent validity of the factors and we determined the outer loadings of the individual trust items. All outer loadings were satisfactorily (they were all above the threshold of 0.5 [31]), except for the item Trust in the treatment 4 (which scored 0.363). Therefore, we removed this item from the measurement model. For the resulting items, we assessed cross loadings. These results can be found in Table 1. All items scored higher on their own factor than on the other factors. In other words, the items assess the factor they are supposed to measure, instead of one of the other factors.

**Table 1:** Item cross loadings

	Latent variable				
	TO	TT	TCT	Ttech	TS
TO 1	**.85**	.42	.37	.23	.20
TO 2	**.90**	.48	.54	.42	.36
TO 3	**.86**	.50	.53	.32	.30
TO 4	**.89**	.50	.53	.22	.23
TO 5	**.90**	.57	.60	.33	.27
TT 1	.51	**.68**	.52	.26	.22
TT 2	.53	**.85**	.52	.41	.30
TT 3	.32	**.72**	.40	.43	.26
TT 5	.37	**.82**	.39	.44	.33
TCT 1	.55	.50	**.85**	.31	.38
TCT 2	.56	.56	**.89**	.41	.45
TCT 3	.39	.43	**.79**	.58	.55
TCT 4	.55	.51	**.88**	.44	.45
Ttech 1	.33	.52	.43	**.70**	.47
Ttech 2	.36	.50	.56	**.81**	.61
Ttech 3	.27	.45	.50	**.83**	.62
Ttech 4	−.03	.13	.13	**.56**	.40
Ttech 5	.22	.28	.32	**.85**	.66
TS 1	.30	.36	.54	.77	**.85**
TS 2	.17	.23	.37	.52	**.78**
TS 3	.22	.19	.34	.58	**.77**
TS 4	.33	.31	.44	.51	**.72**
TS 5	.07	.20	.23	.14	**.50**

Highest loadings are displayed in bold

TO, Trust in Organization; TT, Trust in Treatment; TCT, Trust in Care Team; Ttech, Trust in technology; TS, Trust in Service

Subsequently, we assessed the reliability of the different measurement scales by determining the composite reliability score, the Average Variance Extracted (AVE), and Cronbach’s alpha for each construct (see Table 2). All scores exceed the thresholds for reliable measurement (composite reliability > 0.7, AVE > 0.5, Cronbach’s alpha > 0.7 [31]). We can conclude that the measurement scales are reliable.

**Table 2:** Scale reliability

	Composite reliability	AVE	Cronbach’s alpha
Trust in the organization	.95	.78	.93
Trust in the treatment	.85	.59	.77
Trust in the care team	.92	.73	.88
Trust in technology	.87	.87	.81
Trust in the service	.85	.54	.79

Next, we verified that there was no multicollinearity (discriminant validity) among the constructs that influence the endogeneous factor Trust in the Service by determining Variance Inflation Factors (VIF) scores. These scores were all well below the threshold of 5.00 [31] (Trust in the Organization 1.72. Trust in the Treatment 1.84, Trust in the Care Team 2.03, Trust in the Technology 1.52). Correlations between all factors can be found in Table 3. Only one correlation (between Trust in the technology and Trust in the service) is slightly above the threshold of 0.7 [32]. Next, we considered the square root of the AVE of each construct (Trust in the organization: 0.88, Trust in the treatment: 0.76, Trust in the care team: 0.85, Trust in the technology: 0.93, Trust in the service: 0.74). None of the factor’s square roots of the AVE is lower than its highest correlation with another factor, which is the rule of thumb for ruling out multicollinearity [31]. Taking into account that all scores were good, except for the correlation between Trust in the technology and Trust in the service (which is slightly above the acceptable threshold), we conclude that multicollinearity does not play a role.

**Table 3:** Correlations among latent variables

	TO	TT	TCT	Ttech	TS
TO	1.00				
TT	.58	1.00			
TCT	.64	.60	1.00		
Ttech	.53	.61	.65	1.00	
TS	.39	.35	.56	.74	1.00

TO, Trust in Organization; TT, Trust in Treatment; TCT, Trust in Care Team; Ttech, Trust in technology; TS, Trust in Service

Finally, we assessed the significance and relevance for the individual indicators, with respect to the latent variable they are supposed to measure. To this goal, we used a bootstrapping procedure using 5,000 samples (following the guidelines in [31]), to determine whether the contribution of each item towards its factor is significantly greater than 0. Except for one item (Trust in the Service 5), this was the case. However, as the outer loading for this item was 0.5, and it therefore does add to the measurement of the factor [31], we retained it in the measurement model.

Via these analyses, we were almost fully able to replicate the validation of the PATAT instrument. The only exception being that the item Trust in the Treatment 4 did not contribute to the measurement model. In the subsequent analyses (for determining the causal model) we will not use this item. Considering all the satisfactory validity and reliability scores that we reported, the remaining measurement model can be considered to be good.

### Causal model

**Fig. 3:**
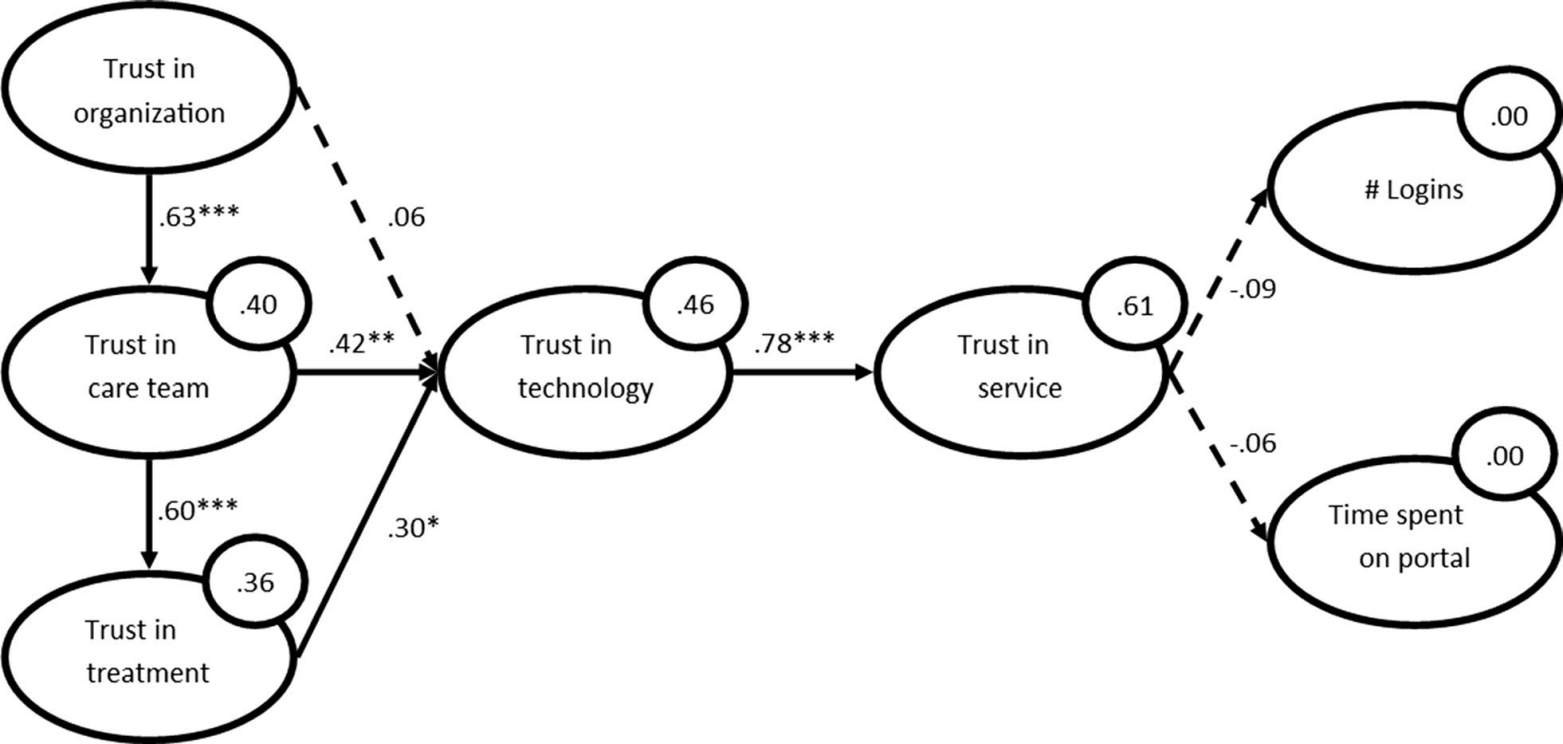
Causal model. *p < .05; **p < .01; ***p < .001

We developed the causal model by means of a bootstrapping procedure with 5000 bootstraps. The result was an overview of path coefficients, their t-values, and significance levels. These scores, together with the explained variance (R^2^) can be found in Fig. 3.

Then we determined the effect size (*f*^2^) of the significant relations in the model by determining the change in R^2^ for the dependent variables when each of its dependent variables is removed from the model. These scores were interpreted according to the guidelines by Cohen [33] (> 0.02 small, > 0.15 medium, > 0.35 large effect size). The changes in R^2^ are as follows:

- Trust in the organization → Trust in the care team: *f*^2^ = 0.66 (large effect size)
- Trust in the care team → Trust in the treatment: *f*^2^ = 0.55 (large effect size)
- Trust in the care team → Trust in the technology: *f*^2^ = 0.17 (medium effect size)
- Trust in the treatment → Trust in the technology: *f*^2^ = 0.09 (small effect size)
- Trust in the technology → Trust in the service: *f*^2^ = 1.59 (large effect size)

## Discussion

In this study, we determined which factors contribute to the coming about of trust in rehabilitation care and whether this trust is transferred to a related eHealth service (in this case, an online patient portal). Next, we studied whether the trust in the online patient portal affected its use by patients. Our results show that, for a rehabilitation care context, trust in the organization has a large impact on trust in the care team, which on its turn, has a large impact in trust in the treatment. And according to our results, transference of trust between trust in offline care and the online patient portal does take place: Trust in the care team and trust in the treatment affected trust in the portal technology. Trust in the technology had a very large effect on a holistic perception of trust in the online service. This holistic trust, however, did not affect the use of the portal. Finally, our application of the PAtient Trust Assessment Tool (PATAT) [26] and the determination of the measurement model, has re-validated the tool. In this regard, we can conclude that the tool is a valid measurement of the different trust factors, once one item (Trust in the Treatment 4) is removed.

This study shows that the formation of trust in healthcare is not unidimensional, but consists of different factors (in our case trust in the care organization, trust in the care team and trust in the treatment). As such, this study reaches the same conclusion as Zheng et al. [23], who concluded that, in a healthcare setting, trust in the care organization and trust in the caregiver are conceptually different, or Graham et al. [34] who found a conceptual difference between trust in the physician and trust in the healthcare system. Previous research on trust in healthcare has mostly focused on interpersonal trust in the physician or nurse [35, 36] and the most widely used scale for assessing patient trust in a physician, the Wake Forest Physician Trust Scale [37], is unidimensional. Likewise, many studies have studied the antecedents of trust in healthcare and its consequences with only one main factor, like trust in the primary care physician [38] or medical specialist [39]. Our study resulted in a measurement model that makes a clear distinction among five trust factors, while our causal model shows that a multitude of factors make up trust in an eHealth service. Researchers should take a more holistic view when studying the coming about of trust and its consequences in healthcare, rather than restricting themselves to one entity only.

Our study shows that trust in the care team and trust in the treatment affect trust in the technology (which can be considered a case of trust transference). Trust in the care organization providing the care does not affect trust in the technology. The latter is somewhat surprising, as this transference has been shown in non-medical contexts [10, 40] and since trust in the organization that offers a technology is an elemental part of the most widely-known model that explains online trust [41]. Our results suggest that trust transference towards trust in the health technology is determined by trust factors with a more interpersonal nature.

In our research model, we hypothesized that trust in the online service would affect the use of the online service. However, the causal relationships between trust in the online service and parameters of use were not significant. This is puzzling, as it undermines the large body of literature that claims that trust in an online service is a prerequisite for use (e.g., [42]). Of course, there is a wide variety of factors that can affect use of such a portal, like motivational cues (stimulation by the care team and/or fellow patients), perceived severity of the disease, general satisfaction with the eHealth service, or the classic technology acceptance factors perceived ease of use and perceived usefulness [43–45]. It could be the case that trust in an online service does not affect use directly, but affects one or more of these antecedents of use. This would be in line with other studies, that found that trust affects (the intention to) use indirectly via satisfaction [46] or perceived ease of use [8]. If we were to choose an explanation for the absence of a significant relation between trust in the online service and use in our study, we would opt for the latter. As we included different types of patients, that received care from a wide range of care professionals, motivational and disease-specific explanations seem unlikely. Due to the design of our research model, however, we cannot test whether trust-related factors affect use indirectly.

Trust plays an important role when implementing a new eHealth service. This study shows that, in order for patients to trust an eHealth service, a care organization should ensure trust in the care team and treatment as well. Since these types of trust mostly originate from personal interaction with care professionals [24], care organizations should create a context that fosters trust. This can be done by making sure that care professionals are perceived as competent and that both the organization and care professionals are transparent about the treatment they provide, and that they are honest and open towards patients [4].

### Limitations

This study knows some limitations. First, the study was conducted within the Netherlands and in the context of rehabilitation care. In the Netherlands, each citizen has a basic healthcare insurance for an affordable fee, thereby limiting the role of healthcare insurers. As a result, trust in insurers will play only a marginal role in the Netherlands, while it will be a major factor in healthcare systems where health insurers do have power (e.g., the United States of America). Additionally, the Dutch healthcare system is considered to be one of the best in Europe [47] and shared-decision making between care professionals and patients is highly promoted. In all, this limits the generalizability of our results. Second, we were not able to test how external events (such as negative news about the use of online services in healthcare, like hackers breaking into systems) influenced patient trust and use of the portal. This would have meant including an additional means of data collection and a complex data analysis plan that takes into account the interference of these events. Finally, we were unable to collect some demographic data (level of education, length of their period of treatment) and were therefore unable to report a full demographical profile of the participant sample.

## Conclusions

Trust has become a pivotal factor with regard to novel eHealth technologies. Technologies that are rapidly developing, and are now able to detect symptom changes at home using unobtrusive sensors, use automated decision support tools with artificial intelligence for tailored treatment plans, offer personalized coaching, and that can anticipate disease progression. It is very likely that such eHealth technologies will (partly) substitute face-to-face care and will take over parts of the decision-making process. The widespread adoption and effect of these innovations will rely on their end-users (patients, care professionals) trusting these novel technologies. It is therefore of paramount importance that we increase our understanding of the concept of trust and make it an integral part of our design and evaluation practices in eHealth. Future work should focus on these challenges by replicating this study for other care domains and other types of eHealth systems (especially those in which Artificial Intelligence plays a large role). Additionally, it would be highly interesting to replicate this study while including the factors Ease of use and Usefulness and to test whether trust-related factors affect use via these additional factors. And finally, longitudinal studies in which the level of trust is benchmarked at regular intervals and that will allow us to study the effect of external events on trust (e.g., data leaks, successful treatment) would be a welcome addition to the body of literature.

## Supplementary Information

**Additional file 1**. Measurement items.

## Data Availability

Data is available upon request from the first author.

## Abbreviations

AVE: Average Variance Extracted
PATAT: PAtient Trust Assessment Tool
PLS-SEM: Partial Least Squares Structural Equation Modelling
TCT: Trust in the Care Team
TO: Trust in the Organization
TS: Trust in the Service
TT: Trust in the Treatment
Ttech: Trust in Technology
VIF: Variance Inflation Factor
