# Supplementary material for: The formation of patient trust and its transference to online health services: the case of a Dutch online patient portal for rehabilitation care
Source: BMC Med Inform Decis Mak. 2021 Jun 12;21:188. doi: 10.1186/s12911-021-01552-4 (PMC8199797; doi:10.1186/s12911-021-01552-4)
Supplement: Supplementary file 1 — Additional file 1. Measurement items. [file 12911_2021_1552_MOESM1_ESM.docx]

Measurement items

Changes to the original PATAT instrument are underlined.

**Trust in the care organization**

1 Roessingh has a good reputation

2 At Roessingh they handle my personal information carefully

3 At Roessingh they take action when something goes wrong

4 At Roessingh, I feel at ease

5 At Roessingh, they take my specific needs into account

**Trust in care professional**

1 I trust my care team’s judgment about my medical care

2 My care team provides me with all the information on all potential medical options

3 My care team keeps all my medical information private

4 I always follow my care team’s advice

**Trust in treatment**

1 The treatment I receive is effective

2 It is clear to me what the treatment I receive entails

3 Together, my care team and I made the choice for this treatment

4 The treatment I receive is not helping me enough

5 It has been explained well to me what my treatment entails

**Trust in technology**

1 When I use the portal, I am in control

2 Everything that I do on the portal remains private

3 The personal information that is stored at the portal will not get lost

4 The portal is easy to use

5 Legal policy and technological safeguards make the portal a safe environment

**Trust in eHealth service**

1 I can trust the portal

2 I can trust that possible problems with the portal will be solved properly

3 I can trust the portal less than other online services, such as Bol.com and the website of my municipality

4 I feel at ease when working with the portal

5 I do not like to enter my personal data on the portal
